# Synthesis, optical properties, DNA, β-cyclodextrin interactions, and antioxidant evaluation of novel isoxazolidine derivative (ISoXD2): A multispectral and computational analysis

**DOI:** 10.1016/j.heliyon.2024.e34561

**Published:** 2024-07-14

**Authors:** Ibrahim A. Alhagri, Raghad Alsowayan, Siwar Ghannay, Sadeq M. Al-Hazmy, Iqrar Ahmad, Harun Patel, Adel Kadri, Kaiss Aouadi

**Affiliations:** a Department of Chemistry, College of Science, Qassim University, Buraidah, 51452, Saudi Arabia; b Department of Chemistry, Faculty of Sciences, Ibb University, Ibb, Yemen; c Department of Chemistry, College of Science, Sana'a University, Sana'a, P.O. Box 1247, Yemen; d Division of Computer Aided Drug Design, Department of Pharmaceutical Chemistry, R. C. Patel Institute of Pharmaceutical Education and Research, Shirpur, 425405, Maharashtra, India; e Faculty of Science and Arts in Baljurashi, Al-Baha University, P.O. Box (1988), Al-Baha, 65527, Saudi Arabia; f Faculty of Science of Sfax, Department of Chemistry, University of Sfax, B.P. 1171, 3000, Sfax, Tunisia; g Department of Chemistry, Laboratory of Heterocyclic Chemistry Natural Product and Reactivity/CHPNR, Faculty of Science of Monastir, University of Monastir, Avenue of the Environment, Monastir, 5019, Tunisia

**Keywords:** DNA, β-Cyclodextrin, ISoXD2, Solvent effect, Physicochemical and spectroscopic methods

## Abstract

**ISoXD2** are well explored among versatile and outstanding class of pharmacophores for the preparation and discovery of drugs. Herein, the electronic absorption and emission spectra of **ISoXD2** were investigated in three different solvents. The observed transition was attributed to π-π* with charge transfer character. Changes in the excited state and shift of the absorption and emission peaks to longer wavelengths are observed as a result of increasing solvent polarity, due to the interactions between the ISoXD2 molecule and the solvent molecules surrounding it. Changing the solvent confirms its solvatochromic effect. UV–vis and fluorescence analysis revealed that **ISoXD2** binds to deoxyribonucleic acid (DNA) by intercalation mode, with a stoichiometric ratio of 1:1.5. Moreover, the fluorescence intensity of DNA bound to ethidium bromide (EB) in the presence of **ISoXD2** was investigated. From this analysis, the Stern-Volmer quenching constant (K_sv_), quenching rate constant (k_q_), binding constant (K_b_) and binding sites number (n) were found to be 5.654 × 10^3^ M^−1^, 2.827 × 10^11^ M^−1^ s^−1^, 3.81 × 10^4^ M^−1^ and 1.225, respectively. The interaction between **ISoXD2** and β-CD was investigated through absorption spectra analysis. K_b_ for this interaction was determined to be 4.9 × 10^4^ M^−1^. The free radical-scavenging ability of the prepared **ISoXD2**, examined by DPPH and ABTS assays have shown a good antioxidant activity. Furthermore, modeling study was conducted to explore their plausible binding mechanism with **ISoXD2** and to support the experimental results.

## 1 Introduction

Interest in the synthesis of isoxazolidine derivatives has become noticeable due to the numerous biological and photochemical activities acquired by these derivatives [[1], [2], [3], [4], [5], [6]] In our previous study [7], we conducted a comprehensive spectral analysis of a single isoxazolidine derivative. When compared to the derivative in ethanol solvent, the absorption spectra of this derivative in water exhibited a blue shift to 340 nm, indicating the presence of hydrogen bonding interactions. Furthermore, in the excited state, we observed a more pronounced effect, where a change in the Stokes shift across different solvents, with an increase corresponding to the relative solvent polarity, was observed. Isoxazolidine derivatives and some coumarin derivatives were used as photochemical sensors for pH and the determination of a trace quantity of metal ions [8]. We found that the deprotonated form exhibited significantly higher emission intensity compared to the protonated form [7,8]. Building upon these findings, the present study focuses on the creation and examination of the optical characteristics of a novel **ISoXD2**.

Recent research in the fields of life science, chemistry, and clinical medicine has focused on studying the interaction between small molecules (referred to as drugs or ligands) and DNA. There are two commonly employed methods for studying the interaction between DNA and drugs: in vivo and in vitro. In vivo studies involve conducting experiments within living organisms or specific biological systems, providing a realistic representation of the complex physiological environment. On the other hand, in vitro studies are performed in controlled laboratory settings outside of living organisms, allowing for precise control over experimental conditions and focused investigations of molecular interactions. Both in vivo and in vitro approaches offer distinct advantages and play complementary roles in advancing our understanding of drug-DNA interactions. It is noteworthy that our study will be limited to in vitro investigations, with an emphasis on ex vivo controlled laboratory settings [[9], [10], [11], [12], [13], [14]] Understanding the interaction between drugs and DNA is of great importance for disease prevention, enhancing the effectiveness of drugs, and designing and screening new medicines [[15], [16], [17]]. Although DNA exhibits natural fluorescence, but the intensity is typically too weak to directly utilize the fluorescence emission of DNA for studying its properties [18]. Fluorescent compounds such as EB and methylene blue are commonly employed as probes to investigate the structure of DNA in interactions involving drugs and proteins [19]. The fluorescence intensity and lifetime of these pigments are significantly enhanced upon binding to double-stranded DNA. EB exhibits a higher affinity for DNA duplex compared to acridine orange and methylene blue, as it offers greater accessibility for DNA binding [[20], [21], [22]]. Therefore, EB was chosen as the probe in this study.

In accordance with our objectives, we propose in this article to design and synthesize a new isoxazolidine derivative, then to study its antioxidant potential, ADMET properties using pkCSM, molecular docking, optical properties, DNA and β-cyclodextrin interactions.

## 2 Results and discussion

### 2.1 Chemistry

**Scheme 1:**
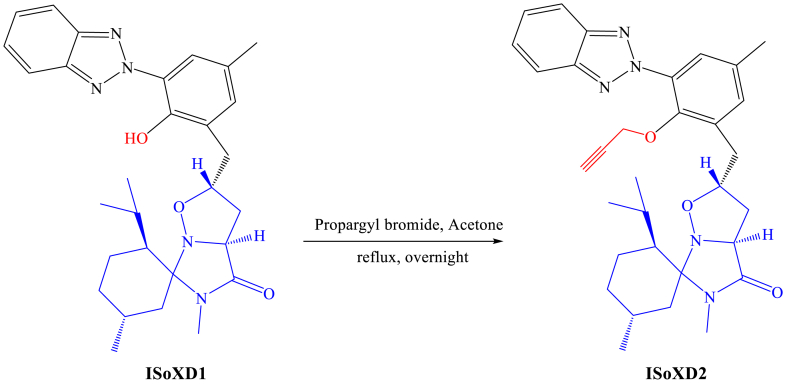
Synthesis of ISoXD2.

The desired product **ISoXD2** was prepared from ISoXD1 [7], itself obtained by 1,3-DC between nitrone **1** and 2-allyl-6-(2H-benzo[d] [[1], [2], [3]] triazol-2-yl)-4-methylphenol [7]. **ISoXD2** was obtained by the alkylation of ISoXD1 with propargyl bromide (Scheme 1).

### 2.2 ISoXD2's UV–Visible and fluorescence spectra

**Fig. 1:**
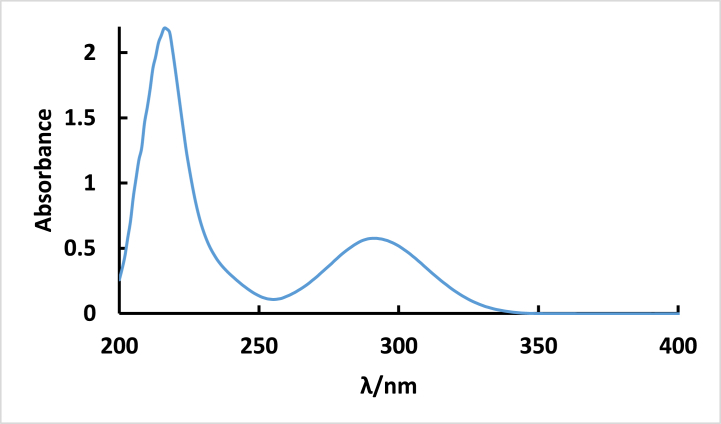
Absorption spectrum of 4.2 × 10^−5^ M ISoXD2 in methanol.

In ethanol, **ISoXD2** displays a pronounced and robust absorbance peak at 216 nm, indicating the spin-allowed S_0_→S_2_ transition. A moderately intense band appearing at a longer wavelength of 290 nm signify a spin-allowed S_0_→ S_1_ transition see Fig. 1 [23]. The observed transition was attributed to π-π* with charge transfer character.

**Fig. 2:**
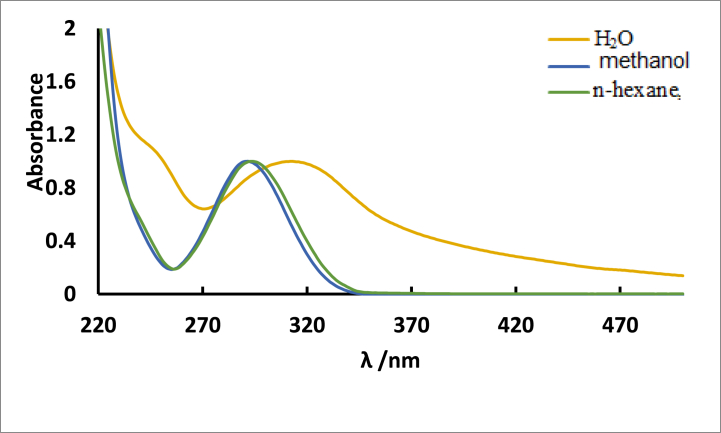
Absorption spectra of 4.2 × 10^−5^ M ISoXD2 in n-hexane, methanol and H_2_O.

The absorption spectra of the substance are relatively constant in n-hexane and methanol solvents due to the insensitivity of initial electronic transitions to changes in the solvent relative polarity indicating a little change in dipole moments on going from ground state to excited state. However, a bathochromic shift is observed when the substance is dissolved in water, and this shift is attributed to the hydrogen bonding interaction between the substance and water molecules as shown in Fig. 2. The appearance of a broad and structureless absorption spectrum of the aqueous solution in comparison to that in methanol, are caused by the interaction of the **ISoXD2** functional groups that are capable of hydrogen bonding with water molecules see Fig. 2 [8].

**Fig. 3:**
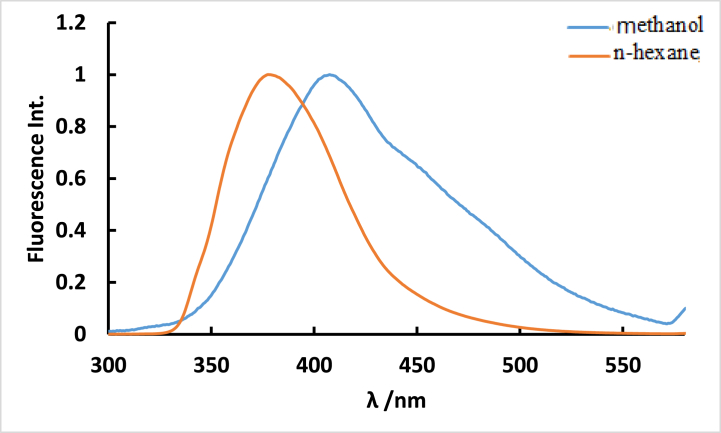
Normalized fluorescence spectra of 4.2 10^−5^ M ISoXD2 in n-hexane and methanol solvents, λ_ex_ = λ_ab_. _max_.

The 30 nm red shift in the emission maximum peak of the **ISoXD2**, despite identical absorption spectra in both regular n-hexane and methanol solvents, is attributed to the solvatochromic effect. This effect is caused by changes in the excited state and interactions between the compound molecule and surrounding solvent molecules, leading to a shift towards longer wavelengths in response to increasing solvent polarities, indicating a higher dipole moment in the excited state than in the ground state as shown in Fig. 3.

**Fig. 4:**
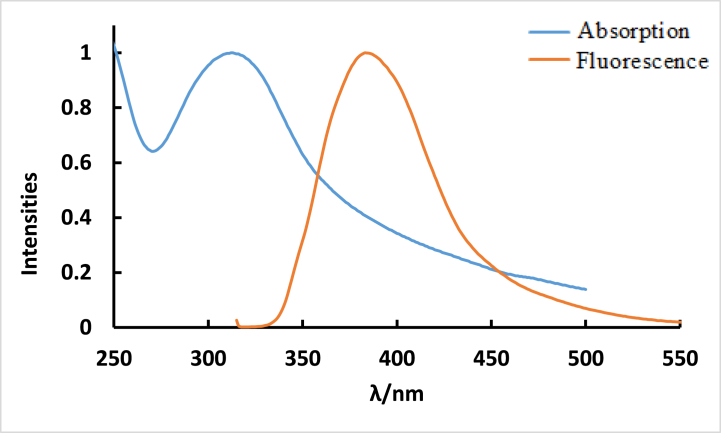
Normalized absorption and fluorescence spectra of 4.2 × 10^−5^ M in H_2_O, λ_ex_ = 291 nm.

Fig. 4 and Table 1 demonstrate the substantial Stokes shift of approximately 116 nm observed for 4.2 × 10^−5^ M **ISoXD2** in methanol. Upon excitation, **ISoXD2** absorbs energy and undergoes a transition to a higher energy state. Subsequently, the molecule engages in vibrational relaxation, which occurs due to molecular interactions and collisions. This relaxation process allows the molecule to release excess energy through vibrations and stabilize in the lowest vibrational state of the first electronically excited state. During this transition, the molecule returns to different vibrational levels in the ground state, leading to a change in the emitted light's wavelength. This phenomenon illustrates the impact of relaxation on the molecule's dipole moment and, consequently, alters the wavelength of the emitted light [24] ‏

**Table 1:** Measured maximum absorption (λ_abs_), emission wavelengths (λ_em_), and stock's shift values for ISoXD2 in 3 different solvents.

Solvents	λ_abs, max_ (nm)	λ_em, max_ (nm)	Relative polarityΔ f	Stock's Shift (nm)	Flu. Int.
n-hexane	294	377	0.0014	83	852.86
CH_3_OH	291	407	0.762	116	58
H_2_O	312	383	1.0000	71	126.02

**Fig. 5:**
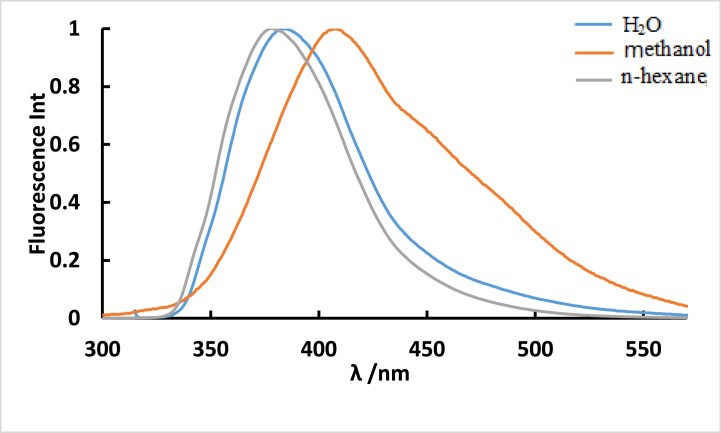
Normalized fluorescence spectra of 4.2 10^−5^ M **ISoXD2** in n-hexane, methanol and H_2_O solvents, λ_ex_ = λ_ab. max_.

From Fig. 5, we notice the red shift in the wavelength of the emission peak of the **ISoXD2** when changing from n-hexane to methanol. This is due to the change in the surrounding electronic environment, which leads to a change in energy levels and a bathochromic shift in the emission wavelength. Contrary to usual, the increase in solvent polarity caused a blue shift in the fluorescence emission maximum of **ISoXD2** in H_2_O by 24 nm compared to methanol as shown also in and Table 1. This may be because the electronic structure of the studied dye may be affected by specific interactions with water molecules, which may lead to this blue shift. This effect is due to the ability of the studied dye to form hydrogen bonds with water, which can change its electronic configuration and affect the energy levels of the molecule, including the excited state, thus affecting the emission wavelength and contributing to the observed blue shift in fluorescence, which confirms the possibility of solvatochromic tuning.

### 2.3 DNA binding studies

#### 2.3.1 UV–vis spectral study

**Fig. 6:**
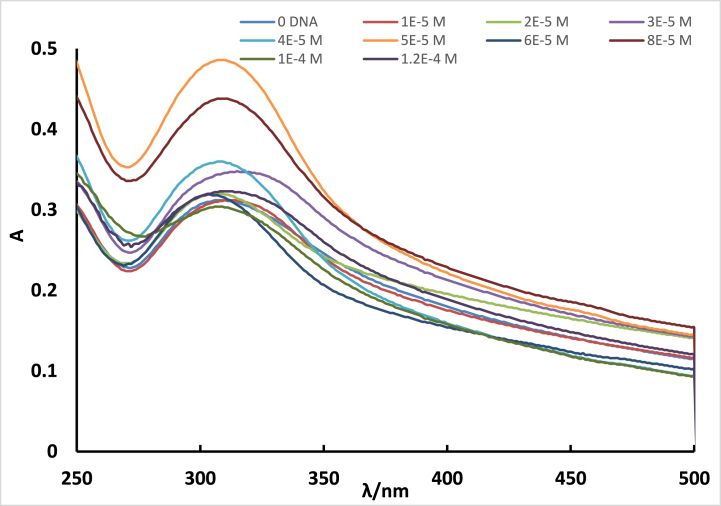
UV–Vis absorption spectra of **ISoXD2** (4 x 10^−5^ M) in the absence and presence of DNA. The cc of DNA from 0 to 10 were (1) 0.0, (2) 10 μM, (3) 20 μM, (4) 30 μM, (5) 40 μM, (6) 50 μM, (7) 60 μM, (8) 80 μM, (9) 100. μM, (10) 120 μM.

The binding mechanism of **ISoXD2** to DNA was investigated through the analysis of absorption spectra. To assess the binding of the **ISoXD2** compound to DNA, various concentrations of DNA were added to the examined molecule. The absorption spectra of the studied dye were captured and depicted in Fig. 6.

**Fig. 7:**
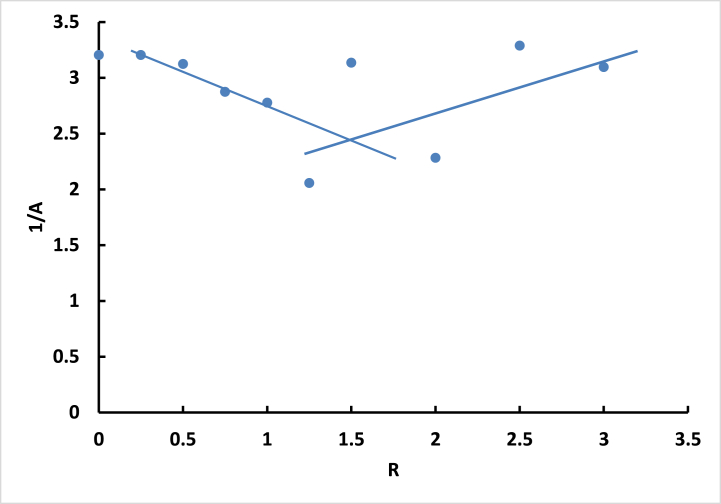
Plot of 1/A versus R for ISoXD2 with DNA

By gradually increasing the amount of DNA added to a fixed quantity of the dye, the absorption intensity gradually increases until the DNA to **ISoXD2** ratio reaches approximately 1:1.5, resulting in a hyperchromic shift (Fig. 7). Beyond this point, the absorption intensity demonstrates irregular fluctuations, characterized by both increases and decreases. Nevertheless, the absorption intensity generally remains higher than that of the free dye. In some DNA additions to **ISoXD2**, a discernible red shift of the wavelength by 9 nm is observed, indicating a bathochromic effect. Additionally, the presence of a hyperchromic effect is suggested by a concomitant increase in absorption intensity, which occurs within a range of concentrations not exceeding a ratio of 1:1.5 of **ISoXD2** to DNA, it can be strongly concluded that the interaction between DNA and **ISoXD2** primarily involves intercalation [25,26].

#### 2.3.2 Fluorescence studies

**Fig. 8:**
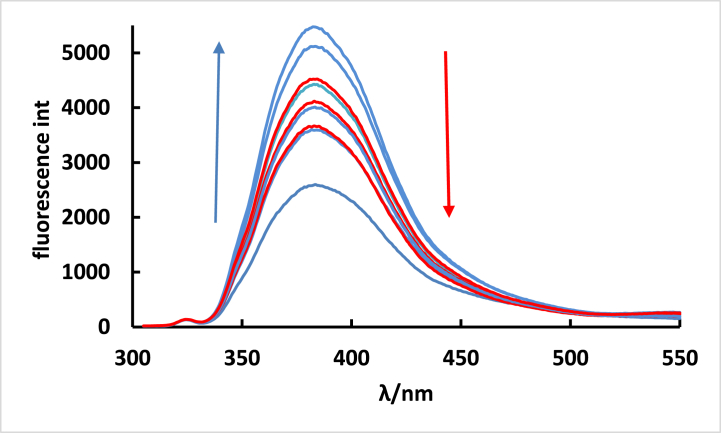
Fluorescence spectra of **ISoXD2** (4 x 10^−6^ M) in the absence and presence of DNA. The cc of DNA from 0 to 9 were (1) 0.0, (2) 1.22 × 10^−6^ M, (3) 2.44 × 10^−6^ M, (4) 3.66 × 10^−6^ M, (5) 4.88 × 10^−6^ M, (6) 6.10 × 10^−5^ M, (7) 7.32 × 10^−6^ M, (8) 8.54 × 10^−6^ M, (9) 9.76 × 10^−6^ M.

Fluorescence spectroscopy proves to be sensitive method for elucidating the intricacies of the interaction and stoichiometry between ISoXD2 and DNA. Employing this technique, the fluorescence properties of **ISoXD2** were examined. It is noteworthy that DNA lacks inherent fluorescence, while **ISoXD2** exhibits pronounced fluorophoric characteristics in aqueous solutions. Specifically, when excited at 292 nm, the emission spectra of ISoXD2 exhibit a broad band centered around 388 nm. (Fig. 8).

Upon the addition of varying amounts of DNA to **ISoXD2**, a continuous enhancement by 109 % in fluorescence intensity of the dye was observed, as depicted by the blue line in Fig. 8. However, following this initial enhancement, a quenching effect became evident, resulting in a reduction in fluorescence intensity. This quenching effect is indicated by the red line in Fig. 8, suggesting a change in the fluorescent properties of **ISoXD2** upon further addition of DNA. The increase in fluorescence signifies that the mode of binding is intercalation [27], which is the conclusion we have reached in UV–vis spectroscopy. Intercalation is characterized by the insertion of a molecule between the base pairs of the DNA double helix, leading to a disturbance in the stacking of the base pairs. The quenching observed when the concentration of DNA exceeds a certain threshold (R ≥ 1.5) is attributed to increased collisions between the fluorophore and DNA, energy transfer or electron transfer processes, and potential conformational changes or alterations in the fluorophore's microenvironment [28].

**Fig. 9:**
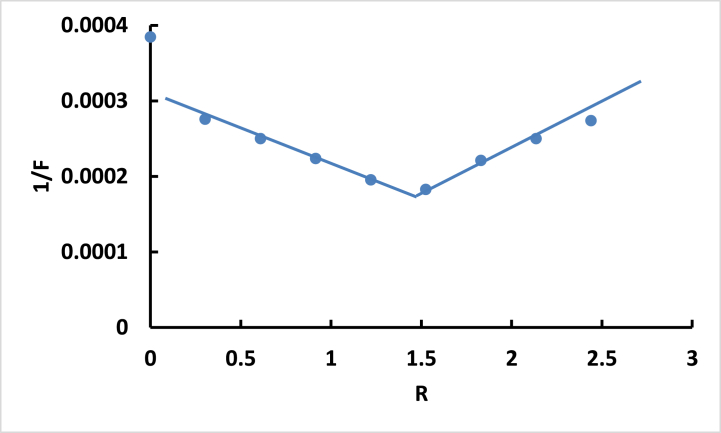
Plot of 1/F versus R for ISoXD2 with DNA

The reciprocal of fluorescence intensity (1/F) plotted against R for -DNA confirm the presence of a 1:1.5 stoichiometric ratio between **ISoXD2** and DNA complexe (Fig. 9), and this is what we obtained from UV–vis spectroscopy [25].

#### 2.3.3 EB–displacement studies

In recent studies, EB has been widely utilized as a probe molecule for investigating the binding of small molecules to DNA. EB binding to DNA is commonly referred to as intercalation binding, which involves the insertion of EB molecules between base pairs in the DNA double helix. EB demonstrates high sensitivity and selectivity, leading to a substantial enhancement in fluorescence intensity upon addition to a DNA solution. This characteristic makes EB a sensitive reagent for determining various properties of DNA.

**Fig. 10:**
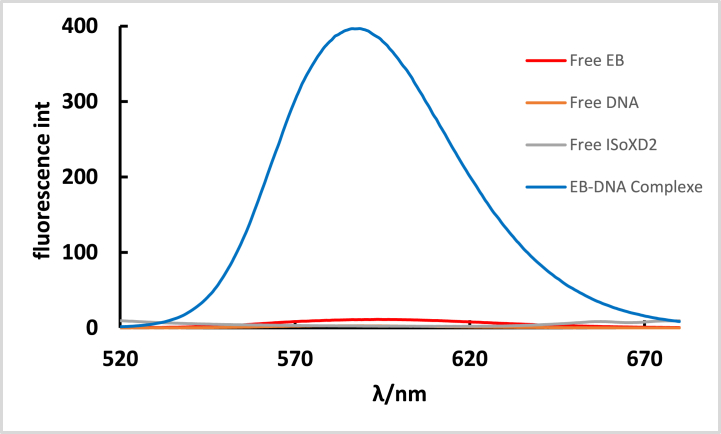
The emission intensity of 2.4 × 10^−5^ M EB, 7.2 × 10^−5^ M DNA, 1.92 × 10^−4^ M ISoXD2 and a mixture of 2.4 × 10^−5^ M EB and 7.2 × 10^−5^ M DNA, at λ_ex_ = 350 nm.

The interaction between the synthesized **ISoXD2** and DNA was investigated through fluorescence quenching experiments using EB. In a Tris buffer solution, free EtBr, free DNA, and **ISoXD2** exhibits extremely weak fluorescence emission in the range of 520–680 nm. In contrast, the mixture of DNA with EB, results in significant fluorescence intensity as shown in Fig. 10.

**Fig. 11:**
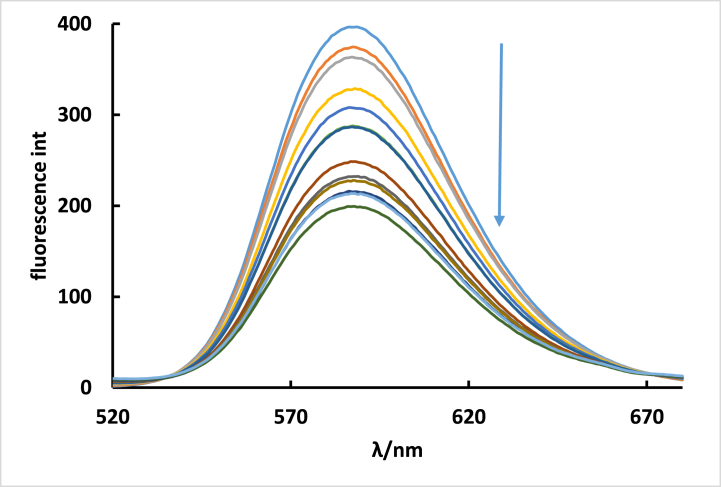
Fluorescence spectra of EB bound to DNA with and without **ISoXD2**, C_EB_ = 2.4 × 10^−5^ M, C_DNA_ = 7.2 × 10^−5^ M; C_ISoXD2_ =(1) 0.00, (2) 1.6 × 10^−5^, (3) 3.2 × 10^−5^, (4) 4.8 × 10^−5^, (5) 6.4 × 10^−5^, (6) 8.0 × 10^−5^, (7) 9.6 × 10^−5^, (8) 1.12 × 10^−4^, (9) 1.28 × 10^−4^, (10) 1.44 × 10^−4^, (11) 1.6 × 10^−4^, (12) 1.76 × 10^−4^ M.

Fig. 11 illustrates the emission spectra of EB bound to DNA, both in the absence and presence of **ISoXD2**. The presence of **ISoXD2**, which inserts itself into the DNA base pair, results in a 50 % decrease in the fluorescence intensity of EB-DNA. This indicates that the interaction between **ISoXD2** and DNA is of the intercalation mode [15].

The fluorescence quenching efficiency is assessed using the Stern-Volmer constant, K_sv_. This constant is determined by applying the classical Stern-Volmer equation [22].

(1) $$\frac{I_{o}}{I}=1+k_{q}\tau_{o}\left[Q\right]=1+k_{sv}\left[Q\right]$$

**Fig. 12:**
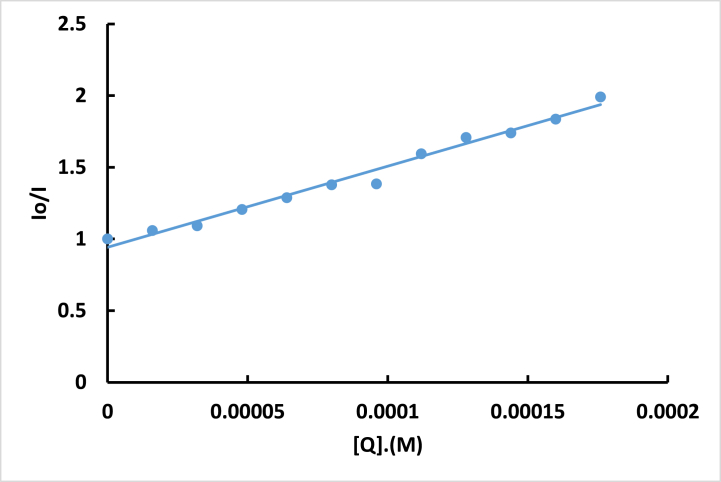
Plot of I/Io versus [Q] for the fluorescence quenching of the EB-DNA complex by **ISoXD2**.

Where Io is the fluorescence intensity without **ISoXD2** and I is the fluorescence intensity in the presence of **ISoXD2**. The DNA-EB quenching rate constant is denoted as K_q_. τ_0_ is the lifetime of DNA–EB without **ISoXD2** and its value is 2 × 10^−8^ S when the ratio between DNA and EB equal to 3 [29]. [Q] represents the concentration of **ISoXD2**. K_SV_ obtained from the slope of the I_0_/I versus [Q] plot. (Fig. 12), and its value is found to be 5.654 × 10^3^ M^−1^. k_q_, is calculated by dividing K_sv_ by τ_0_, resulting in a value of 2.827 × 10^11^ M^−1^ s^−1^. This value is significantly higher than the maximum scatter collision quenching constant observed for various quenchers with biomolecules (2.0 × 10^10^ M^−1^ s^−1^). Consequently, this finding suggests that the interaction between **ISoXD2** and DNA is characterized by static quenching [30,31].

The binding constant (K_b_) and the binding site number (n) of **ISoXD2** with the DNA-EB complex were evaluated using the following equation [22,30]:

(2) $$\log\frac{I_{o}-I}{I}=\log\;k_{b}+n\;\log\left[Q\right]$$

**Fig. 13:**
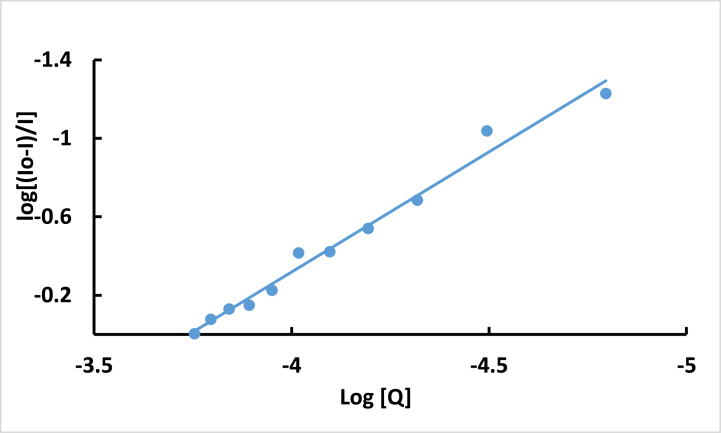
Plot of -log (I_o_-I)/I versus log[Q].

A good linear correlation between log [(I_0_ - I)/I] and log [Q] (Fig. 13) determined K_b_ of 3.81 × 10^4^ M^−1^ and a binding sites number (n) of 1.225.

### 2.4 Host–guest interactions of ISoXD2 with β-CD

**Fig. 14:**
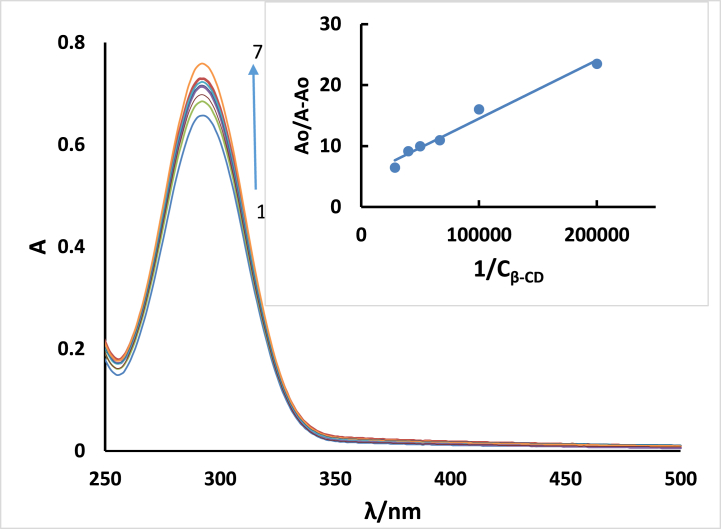
UV–Vis absorption spectra of ISoXD2 (4 x 10-5 M) with and without β-CD. The cc of β-CD from 0 to 5 were (1) 0.0, (2) 0,5 × 10^−5^ M, (3) 1 × 10^−5^ M, (4) 1.5 × 10^−5^ M, (5) 2x10^−5^ M, (6) 2.5 × 10^−5^ M, and (7) 3.5 × 10^−5^ M.

UV–vis spectrophotometric studies are frequently utilized in host-guest supramolecular chemistry to assess the stability of inclusion complexes. When a guest molecule is encapsulated within the cavity of a host molecule, significant changes in the spectral characteristics are observed. By analyzing the UV–vis spectra, researchers can investigate the spectral changes associated with the formation of inclusion complexes and determine the stability constants of these complexes [32,33]. Fig. 14 illustrates the absorption spectra of **ISoXD2** in the presence of different concentrations of aqueous β-CD. As the concentration of β-CD increases, there is a corresponding increase in the absorbance of **ISoXD2**. This suggests that the hydrophobic interior of β-CD effectively accommodates the hydrophobic regions of **ISoXD2**. Furthermore, the absence of a wavelength shift indicates that the electronic transitions of **ISoXD2** remain mostly unaffected by the formation of the complex.

The apparent k_b_ of the inclusion complex can be determined by the following equation:

(3) $$\frac{Ao}{A-Ao}=\frac{\varepsilon_{G}}{\varepsilon_{H-G}-\varepsilon_{G}}+\frac{\varepsilon_{G}}{\varepsilon_{H-G}-\varepsilon_{G}}\times \frac{1}{k_{b}}\frac{1}{C_{\beta -CD}}$$

In which A^0^ and A are the absorption spectrum at *λ*_max_ of the free and complex respectively. The plot of A_0_/A-A_0_ against 1/C_β-CD_ determined the K_b_ value as 4.9 × 10^4^ M^−1^ using the slope and intercept.

### 2.5 Free radical-scavenging ability by the DPPH and ABTS assays

To evaluate the antioxidant potential of the designed analogue, in vitro DPPH and ABTS radical scavenging assays were undertaken. The antioxidant activity was determined in 0–12 mg/mL by increment of 2. The obtained results are depicted in Table 2. As shown, the percentage of inhibitions towards DPPH and ABTS assays increased with concentration with the high level obtained at 12 mg/mL, were around 75.25 % and 80.28 %, and were significantly different (p ≤ 0.05) with the positive controls, ascorbic acid (89.67 ± 4.4 %) and Trolox (90.21 %), respectively. The scavenging ability of **ISoXD2** was determined via IC_50_, and the results revealed good DPPH scavenging capacity of **ISoXD2** with IC_50_ = 8.58 ± 0.36 μM, significantly (p ≤ 0.05) different from ascorbic acid (IC_50_ = 6.03 ± 0.28), and high potency activity against ABTS test with IC_50_ = 9.45 ± 0.44 μM which is not significantly (p ≥ 0.05) different from Trolox (IC_50_ = 9.31 ± 0.43).

**Table 2:** DPPH and ABTS antioxidant activity of the synthesized analogue.

	C (mg/mL)							IC_50_ (μM)
	0	2	4	6	8	10	12	
**DPPH**	0	33.28 ± 1.66^a^	46.69 ± 1.9^a^	52.91 ± 2.3^a^	60.55 ± 3.0^a^	69.35 ± 3.3^a^	75.25 ± 3.4^a^	8.58 ± 0.36^a^
**ABTS**	0	38.23 ± 1.8^a^	48.28 ± 2.02^a^	54.87 ± 2.25^a^	62.14 ± 3.0^a^	70.68 ± 3.25^a^	80.28 ± 3.33^a^	9.45 ± 0.44^a^
**Ascorbic acid**	0	57.34 ± 2.8^b^	66.5 ± 3.1^b^	70.24 ± 3.25^b^	76.78 ± 3.36^b^	84.58 ± 4.2^b^	89.67 ± 4.4^b^	6.03 ± 0.28^a^
**Trolox**	0	61.25 ± 2.9^b^	66.52 ± 3.1^b^	71.22 ± 3.21^b^	76.56 ± 3.34^b^	82.15 ± 4.0^b^	90.21 ± 4.29^b^	9.31 ± 0.43^a^

### 2.6 ADMET study

The main objective of this study is to verify whether the used analogue with specific biological and pharmacological features would be an orally active drug in the human body via its ADMET properties [[34], [35], [36]]. As depicted in Table 3, **ISoXD2** displayed poorly intestinal absorption (96.746 %) and moderate skin permeability. The predicted distribution parameters revealed moderate VDss (extent of drug distribution) and blood–brain barrier (BBB) without fraction unbound. As shown, the cytochrome P450s isoenzymes are crucial for metabolism-based drug–drug interactions, suggest no inhibition for CYP2D6. The prediction of the toxicity level of RS6 indicate good profile with no toxicity.

**Table 3:** ADMET properties of the **ISoXD2** using pkCSM.

Entry	ISoXD2	Reference
**Absorption**		
Water solubility	−5.776	–
Caco2 permeability	1.366	>0.9
Intestinal absorption (human)	96.746	<30 % is poorly
Skin Permeability (log Kp)	−2.746	>-2.5 is low
P-glycoprotein substrate	No	–
**Distribution**		
VDss (human)	0.281	Low is < -0.15, High is > 0.45
Fraction unbound (human)	0	–
BBB permeability	−0.854	Poorly is < -1, High is > 0.3
CNS permeability	−2.125	Penetrate is > -2, Unable is < -3
**Metabolism**		
CYP2D6 substrate	No	No
CYP3A4 substrate	Yes	–
CYP1A2 inhibitior	No	No
CYP2C19 inhibitior	Yes	No
CYP2C9 inhibitior	Yes	No
CYP2D6 inhibitior	No	No
CYP3A4 inhibitior	Yes	No
**Excretion**		
Total Clearance	0.176	–
Renal OCT2 substrate	No	–
**Toxicity**		
AMES toxicity	No	No
hERG I inhibitor	No	No
Oral Rat Acute Toxicity (LD50)	2.986	–
Oral Rat Chronic Toxicity (LOAEL)	1.671	–
Skin Sensitisation	No	No

### 2.7 Molecular docking

**Fig. 15:**
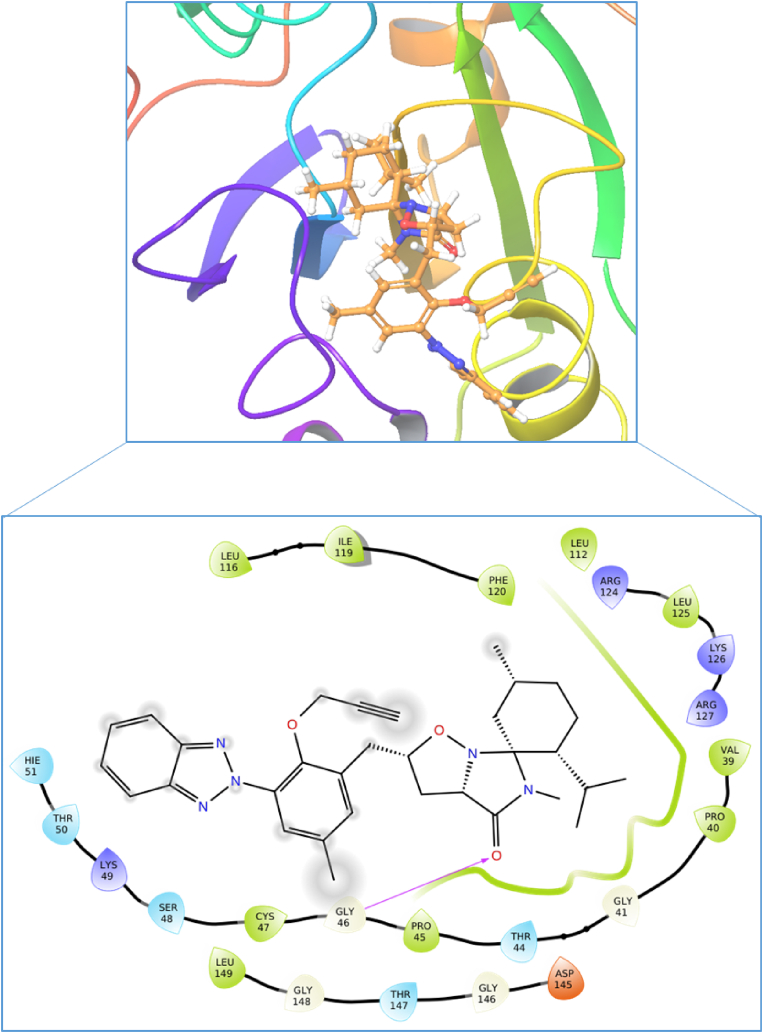
2D and 3D binding interaction of compound **ISoXD2** in the binding cavity of Human peroxiredoxin 5 (PDB ID: 1HD2).

In this study, the antioxidant activity of the **ISoXD2** compound was investigated, and to further elucidate its potential therapeutic relevance, a molecular docking study was conducted on Human peroxiredoxin 5 and DNA. Peroxiredoxin 5, a crucial member of the peroxiredoxin family, plays a significant role in cellular defense against oxidative stress. The docking study aims to explore the binding interactions between **ISoXD2** and the active site of peroxiredoxin 5, shedding light on the molecular mechanisms underlying the compound's antioxidant activity. Insights gained from this investigation may provide valuable information for understanding the compound's potential as a therapeutic agent in combating oxidative stress-related disorders. In the molecular docking study, **ISoXD2** demonstrated a docking score of −5.651 kcal/mol, indicating a favorable binding affinity to the target protein, Human peroxiredoxin 5. In comparison, the co-crystal inhibitor, benzoic acid, exhibited a more favorable docking score of −7.245 kcal/mol, highlighting its higher predicted binding affinity. The binding interaction of **ISoXD2** is characterized by a crucial hydrogen bond formation with Gly46, underscoring a specific and directional interaction within the active site of peroxiredoxin 5 (Fig. 15).

**Fig. 16:**
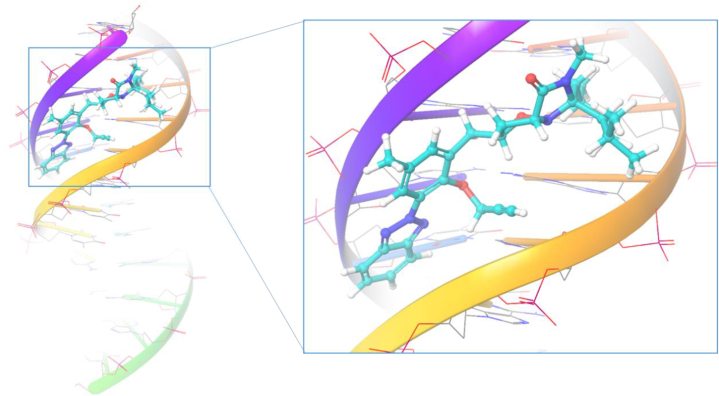
Binding mode of compound **ISoXD2** inside the active site of DNA.

Furthermore, **ISoXD2** establishes van der Waals interactions with a network of surrounding residues, including Hie51, Thr50, Lys49, Ser48, Cys47, Pro45, Thr44, Gly41, Pro40, Val39, Arg127, Lys126, Leu125, and Arg124. These diverse van der Waals interactions collectively contribute to the overall stability of the **ISoXD2** binding within the protein's active site. Notably, Cys47, a cysteine residue common to all peroxiredoxins, is involved in peroxide catalysis in the human peroxiredoxin family. The active pocket, comprising conserved amino acid residues such as Thr44, Gly46, Cys47, and Arg127, facilitates docked chemical identification through hydrogen bonding and hydrophobic interactions. Comparatively, the binding interactions of the co-crystal Benzoic Acid involve Cys47, Thr44, Gly46, Thr147, Pro40, Pro45, Phe120, Arg127, and Leu149. The comparable hydrogen bonding and binding interactions of **ISoXD2** suggest its potential as a promising compound with antioxidant properties, substantiating its candidacy for further exploration in therapeutic applications targeting oxidative stress-related conditions. To corroborate and provide a rational explanation for the experimental findings, an in-depth analysis of the interaction between compound **ISoXD2** and DNA (PDB ID: 1BNA) was also undertaken. Remarkably, during the docking process, compound **ISoXD2** demonstrated a noteworthy minimum binding energy of −4.850 kcal/mol when interacting with DNA. Furthermore, the comprehensive molecular docking studies not only supported but unequivocally confirmed that the binding mode of the complex to DNA is intercalative. In this context, compound **ISoXD2** was found to bind specifically to the helical structure of DNA, exhibiting a distinct intercalative binding pattern as shown in Fig. 16.

## 3 Materials and methods

### 3.1 Synthesis of ISoXD2

To a solution of **ISoXD1** in acetone we add 2 eq of propargyl bromide and 2.5 eq of K_2_CO_3_. The mixture was stirred at reflux for overnight. The solvent was evaporated, and the residue was dissolved in a CH_2_Cl_2_(DCM)/H_2_O mixture. The aqueous phase was extracted with DCM (twice) and the combined organic phases are dried with MgSO4, filtered and evaporated. The **ISoXD2** was obtained after purification on a silica gel column (8/2 Cyclohexane/EtOAc).

^1^H NMR (CDCl_3_,400 MHz) 0.73 (s, 3H, *J* 6.4 Hz, CH_3_); 0.84 (s, 3H, *J* 6.8 Hz, CH_3_); 0.87 (s, 3H, *J* 6.8 Hz, CH_3_); 0.85–0.87 (m, 1H); 1.14 (t, 1H, *J* 12.4 Hz); 1.60–1.63 (m, 3H); 1.72–1.82 (m, 3H); 1.94–1.99 (m, 1H); 2.34 (s, 1H); 2.37 (s, 3H, CH_3_); 2.70 (s, 3H, NCH_3_); 2.72–2.75 (m, 1H); 2.82 (dd, 1H, J 8.8 and 14.0 Hz); 3.30 (dd, 1H, J 3.6 and 13.6 Hz); 4.01 (d, 1H, *J* 8.4 Hz); 4.04–4.09 (m, 1H); 4.11 (d, 1H, *J* 6.8 Hz); 4.18 (dq, 2H, J 2.4 and 13.6 Hz); 7.29 (d, 1H, *J* 1.2 Hz); 7.44 (d, 1H, *J* 3.2 Hz); 7.45 (d, 1H, *J* 3.2 Hz); 7.50 (d, 1H, *J* 1.6 Hz); 7.96 (d, 1H, *J* 2.8 Hz); 7.80 (d, 1H, *J* 3.2 Hz).

^13^C NMR (CDCl_3_,75 MHz) 14.1, 18.4, 20.7, 21.0, 22.1, 22.3, 24.1, 24.3, 26.1, 29.6, 33.3, 34.6, 38.8, 40.4, 48.4, 60.4, 61.4, 66.2, 76.0, 77.2, 78.1, 89.8, 118.43, 125.96, 127.2, 133.4, 133.6, 134.2; 134.5, 145.0, 147.2, 172.7.

Anal. Calcd. for C_32_H_39_N_5_O_3_ (541.69): C, 70.95; H, 7.26; N, 12.93, Found: C, 70.66; H, 7.29; N, 13.02.

### 3.2 UV–vis absorption and fluorescence spectra

A Shimadzu UV–Vis 1650-PC spectrophotometer was used to record UV–Vis electronic absorption spectra. A steady-state fluorescence spectrum was displayed using a quartz cuvette with a 1 cm path length; the emission was observed at a 90° geometry with a Jasco FP-8200 spectrofluorometer that had a 5 nm excitation and emission bandwidth, and it was illuminated with a 5 nm Xenon Lamp light source.

### 3.3 DNA and β-CD binding studies

DNA, β-CD, Ethidium Bromide and tris-(hydroxymethyl)aminomethane (Tris) were obtained from Sigma, and no additional purification steps were performed. To prepare the stock solutions, both β-CD and DNA were dissolved in deionized water. The concentration of the DNA stock solution was determined by measuring its UV absorbance at 260 nm, utilizing a molar extinction coefficient (*ε*) of 6600 M^−1^cm^−1^ [37]. The absence of protein contamination in the DNA sample was confirmed by examining the A_260_/A_280_ ratio, which was found to be 1.89, exceeding the threshold of 1.8. A 1 mM solution of Ethidium Bromide was prepared using deionized water and stored in a light-protected container. The experiments involving ISoXD2 with DNA were conducted using a 10 mM Tris-buffer solution prepared in deionized water. The pH of the solution was adjusted to 7.4 using HCl. For the experiments involving **ISoXD2** with β-CD, a 10 mM Tris-buffer solution with a pH of 7.4 was mixed with ethanol in a 1:1 ratio.

### 3.4 Scavenging activity of ABTS radical dot + free radical

The radical scavenging ability of the tested compound was carried out spectrophotometrically using ABTS^.+^ cation decolorization assay described previously [1,2,[38], [39], [40]]. Trolox was used as reference and all measurements were carried out on three identical samples.

### 3.5 ADMET property predictions

The prediction of the pharmacokinetic parameters such as absorption, distribution, metabolism, elimination and toxicity (ADMET) has been carried out online via pkCSM software, https://biosig.lab.uq.edu.au/pkcsm/accessed on December 17, 2023.

### 3.6 Molecular docking

Molecular docking simulations were performed using the Glide software within the crystal structure of Human peroxiredoxin 5 (PDB ID: 1HD2) and DNA (PDB ID: 1BNA). The docking experimental details, were precisely followed as reported in previous study [[41], [42], [43]].

## 4 Conclusion

Changing the solvent confirms the solvatochromic nature of **ISoXD2**, allowing it to emit light over a variety of wavelengths. Binding of **ISoXD2** to DNA induces a hyperchromic effect and a red shift in the UV-absorption spectra. This binding also leads to a significant increase (>109 %) in fluorescence intensity without a shift in the emission wavelength. These spectral characteristics provide strong evidence for the intercalation of ISoXD2 into DNA. The stoichiometric ratio between **ISoXD2** and DNA complexes was 1:1.5. Furthermore, **ISoXD2** displaces the intercalated EB from the DNA-EB complex. The binding constant of **ISoXD2** to β-CD was determined to be 4.9 × 10^4^ M^−1^, indicating a relatively strong interaction. The scavenging activity of **ISoXD2** against DPPH and ABTS have shown very potent antioxidant property. Modeling evidence explicitly support and confirm that the binding mode of the compound to DNA is intercalated which enhances our understanding of the molecular interactions between the studied compounds and DNA, contributing valuable insights to the broader context of molecular biology and drug discovery.

## CRediT authorship contribution statement

**Ibrahim A. Alhagri:** Writing – review & editing, Writing – original draft, Validation, Methodology, Investigation, Formal analysis, Data curation, Conceptualization. **Raghad Alsowayan:** Visualization, Formal analysis, Data curation. **Siwar Ghannay:** Validation, Software, Conceptualization. **Sadeq M. Al-Hazmy:** Writing – review & editing, Writing – original draft, Methodology, Investigation, Conceptualization. **Iqrar Ahmad:** Writing – original draft, Software. **Harun Patel:** Software. **Adel Kadri:** Writing – review & editing, Writing – original draft, Validation, Supervision, Software. **Kaiss Aouadi:** Writing – review & editing, Writing – original draft, Visualization, Validation, Supervision, Software, Methodology, Conceptualization.

## Declaration of competing Interest

The authors declare that they have no known competing financial interests or personal relationships that could have appeared to influence the work reported in this paper.
