# Supplementary material for: Synthesis, optical properties, DNA, β-cyclodextrin interactions, and antioxidant evaluation of novel isoxazolidine derivative (ISoXD2): A multispectral and computational analysis
Source: Heliyon. 2024 Jul 14;10(14):e34561. doi: 10.1016/j.heliyon.2024.e34561 (PMC11305329; doi:10.1016/j.heliyon.2024.e34561)
Supplement: Multimedia component 1 [file mmc1.pdf]

Supporting information

Synthesis, optical properties, DNA, β-cyclodextrin interactions, and antioxidant evaluation of novel isoxazolidine derivative (ISoXD2): A multispectral and computational analysis

Ibrahim A. Alhagri [a,b], Raghad Alsowayan [a], Siwar Ghannay[a], Sadeq M. Al-Hazmy [a,c], Iqrar Ahmad[d], Harun Patel[d], Adel Kadri[e,f], Kaiss Aouadi[a,g,*]

[a] Department of Chemistry, College of Science, Qassim University, Buraidah 51452, Saudi Arabia
[b] Department of Chemistry, Faculty of Sciences, Ibb University, Ibb, Yemen
[C] Department of Chemistry, College of Science, Sana'a University, Sana'a P.O. Box 1247, Yemen
[d] Division of Computer Aided Drug Design, Department of Pharmaceutical Chemistry, R. C. Patel Institute of Pharmaceutical Education and Research, Shirpur 425405, Maharashtra, India
[e] Faculty of Science and Arts in Baljurashi, Al-Baha University, P.O. Box (1988). Al-Baha 65527, Saudi Arabia.
[f] Faculty of Science of Sfax, Department of Chemistry, University of Sfax, B.P. 1171, 3000 Sfax, Tunisia.
[g] Department of Chemistry, Laboratory of Heterocyclic Chemistry Natural Product and Reactivity/CHPNR, Faculty of Science of Monastir, University of Monastir, Avenue of the Environment, Monastir 5019, Tunisia

* Correspondence: Pr. Kaiss Aouadi, email: K.AOUADI@qu.edu.sa

1H NMR spectrum for ISoXD2 2

13C NMR spectrum for ISoXD2 3

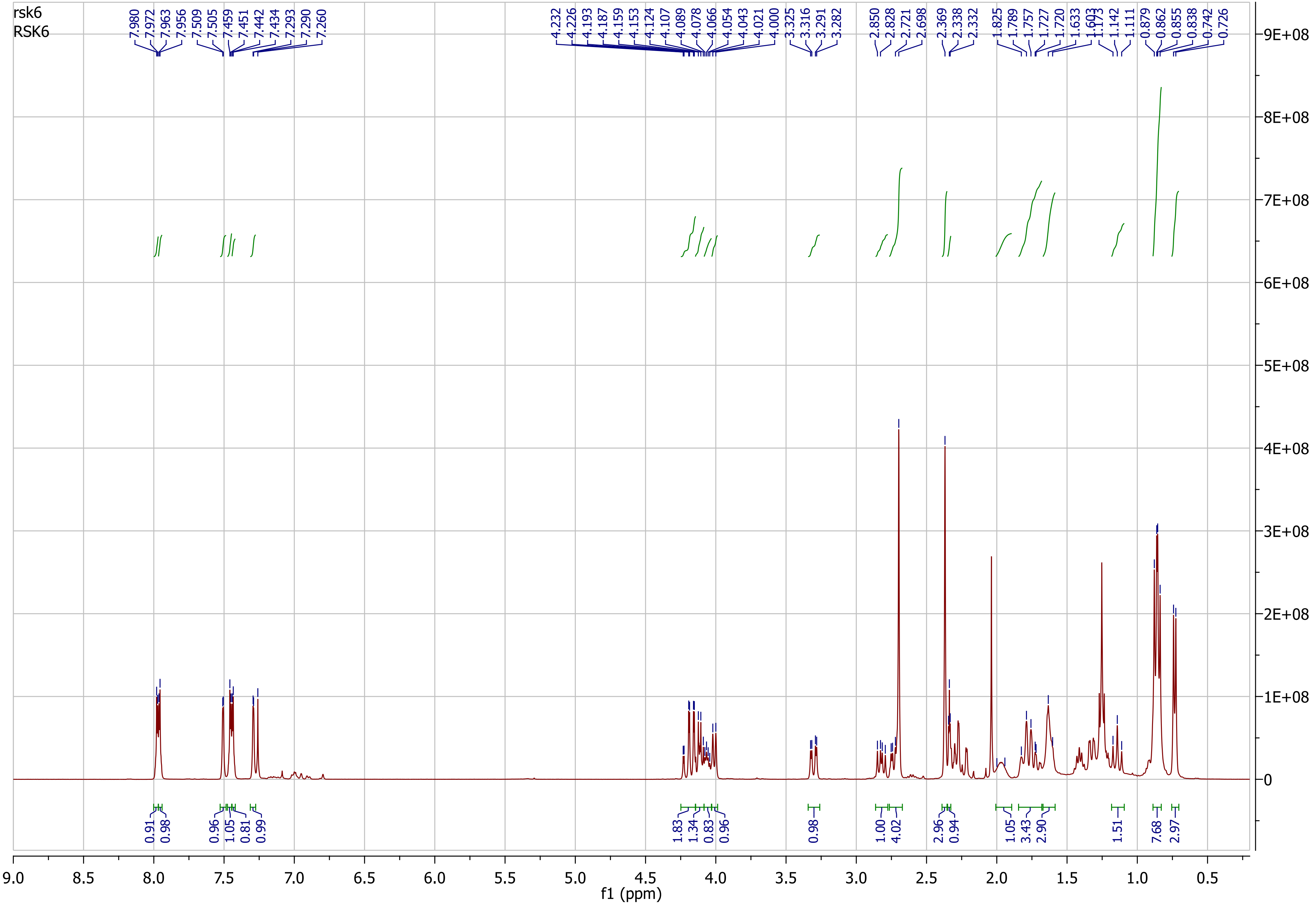
rsk6
RSK6
7.980
7.972
7.963
7.956
7.509
7.505
7.459
7.451
7.442
7.434
7.293
7.290
7.260
4.232
4.226
4.193
4.187
4.159
4.153
4.124
4.107
4.089
4.078
4.066
4.054
4.043
4.021
4.000
3.325
3.316
3.291
3.282
2.850
2.828
2.721
2.698
2.369
2.338
2.332
1.825
1.789
1.757
1.727
1.720
1.633
1.603
1.173
1.142
1.111
0.879
0.862
0.855
0.838
0.742
0.726
0.91
0.98
0.96
1.05
0.81
0.99
1.83
1.34
0.83
0.96
0.98
1.00
4.02
2.96
0.94
1.05
3.43
2.90
1.51
7.68
2.97
9E+08
8E+08
7E+08
6E+08
5E+08
4E+08
3E+08
2E+08
1E+08
0
9.0
8.5
8.0
7.5
7.0
6.5
6.0
5.5
5.0
4.5
4.0
3.5
3.0
2.5
2.0
1.5
1.0
0.5
f1 (ppm)

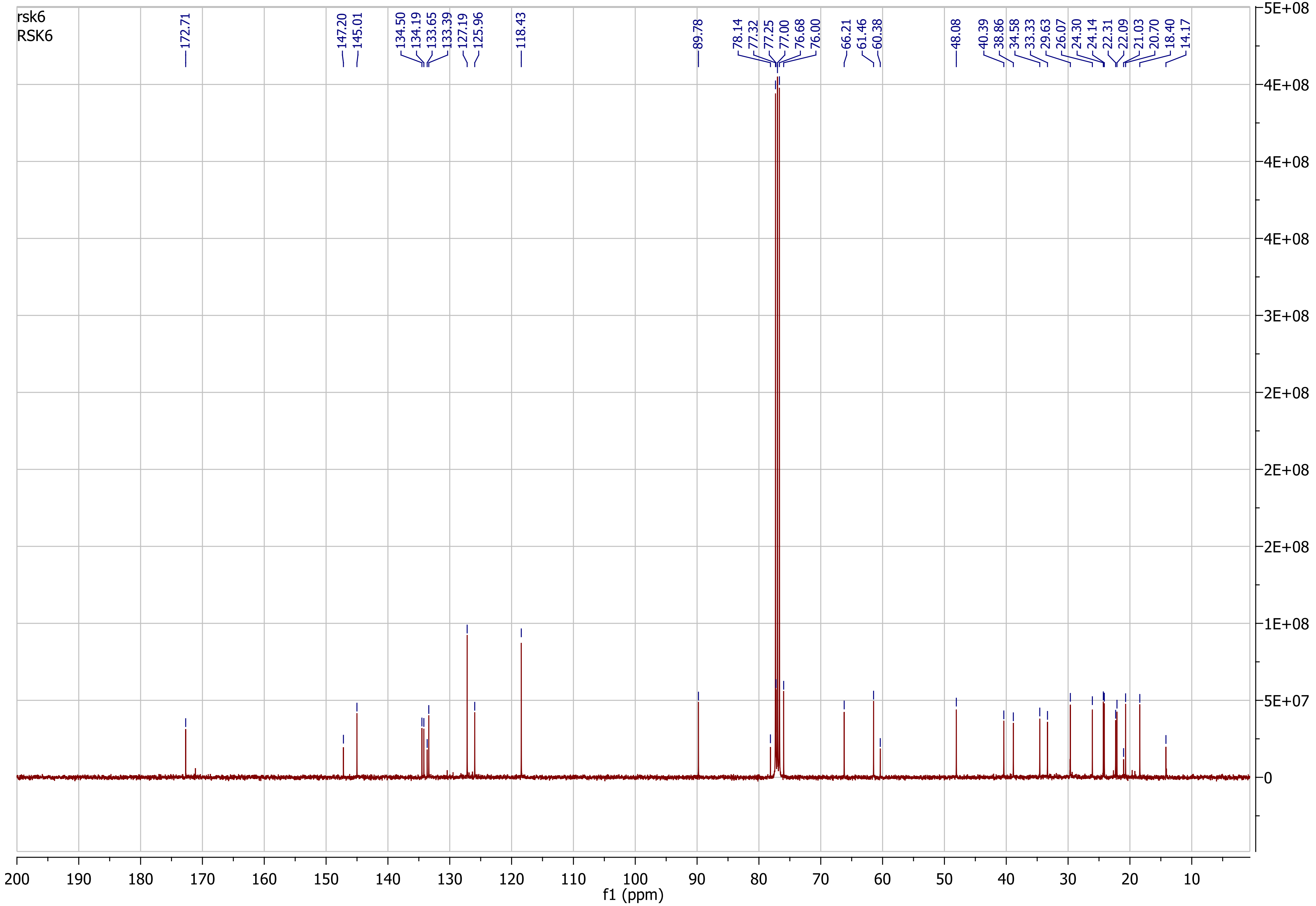
rsk6
RSK6
172.71
147.20
145.01
134.50
134.19
133.65
133.39
127.19
125.96
118.43
89.78
78.14
77.32
77.25
77.00
76.68
76.00
66.21
61.46
60.38
48.08
40.39
38.86
34.58
33.33
29.63
26.07
24.30
24.14
22.31
22.09
21.03
20.70
18.40
14.17
5E+08
4E+08
4E+08
4E+08
3E+08
2E+08
2E+08
2E+08
1E+08
5E+07
0
200
190
180
170
160
150
140
130
120
110
100
90
80
70
60
50
40
30
20
10
f1 (ppm)
